# MicroRNA profiling of Chinese primary glioblastoma reveals a temozolomide-chemoresistant subtype

**DOI:** 10.18632/oncotarget.3258

**Published:** 2015-03-23

**Authors:** Wei Yan, Yanwei Liu, Pei Yang, Zheng Wang, Yongping You, Tao Jiang

**Affiliations:** ^1^ Beijing Neurosurgical Institute, Capital Medical University, Beijing, PR China; ^2^ Department of Neurosurgery, First Affiliated Hospital of Nanjing Medical University, Nanjing, PR China; ^3^ Beijing Institute for Brain Disorders, Brain Tumor Center, Beijing, PR China; ^4^ Department of Neurosurgery, Beijing Tiantan Hospital, Capital Medical University, Beijing, PR China

**Keywords:** glioblastoma, microRNA, IDH1 mutation, temozolomide

## Abstract

Accumulating evidence demonstrates that defining molecular subtypes based on objective genetic alterations may permit a more rational, patient-specific approach to molecular targeted therapy across various cancers. The objective of this study was to subtype primary glioblastoma (pGBM) based on MicroRNA (miRNA) profiling in Chinese population. Here, miRNA expression profiles from 82 pGBM samples were analyzed and 78 independent pGBM samples were used for qRT-PCR validation. We found that two distinct subgroups with different prognosis and chemosensitivities to temozolomide (TMZ) in Chinese pGBM samples. One subtype is TMZ chemoresistant (termed the TCR subtype) and confers a poor prognosis. The other subtype is TMZ-chemosensitive (termed the TCS subtype) and confers a relatively better prognosis compared with the TCR subtype. A classifier consisting of seven miRNAs was then identified (miR-1280, miR-1238, miR-938 and miR-423-5p (overexpressed in the TCR subtype); and let-7i, miR-151-3p and miR-93 (downregulated in the TCR subtype)), which could be used to assign pGBM samples to the corresponding subtype. The classifier was validated using both internal and external samples. Meanwhile, the genetic alterations of the TCR and TCS subtypes were also analyzed. The TCR subtype was characterized by no IDH1 mutation, and EGFR and Ki-67 overexpression. The TCS subtype displayed the opposite situation. Taken together, the results indicate a distinct subgroup with poor prognosis and TMZ-chemoresistance.

## INTRODUCTION

Glioblastoma (GBM), which is the most lethal glioma, often proves intractable to traditional cancer treatments such as surgery, chemotherapy and radiation, and quickly invades healthy brain tissue [1]. Biotherapy and molecular targeted therapy are thought to be breakthroughs in the future treatment of glioma [2]. However, the current grading system, based on histopathological diagnosis, cannot provide details for biotherapy and molecular targeted therapy and has been associated with significant intraobserver variability. Moreover, the etiology underlying the development of biotherapy and molecular targeted therapy is unclear. Thus, classification based on gene profiles may offer an objective subtype classification system, reveal the underlying molecular mechanisms and help to identify subtype or even patient-specific targets for biotherapy and molecular targeted therapy. To date, classifications of glioma based on messenger RNA (mRNA) expression data have been attempted in the past, with varying success and with only some concordance between studies [3–6]. Moreover, mRNA is labile and prone to degradation. The bias caused by degradation often affects the results of classification. Thus, the outlook for the clinical use of classification based on mRNA expression profiles is poor.

MicroRNAs (miRNAs) are a class of small non-coding RNA molecules that regulate the expression of multiple target genes, affecting multiple cellular processes including cell differentiation, stem cell maintenance, and epithelial–mesenchymal transition. The abnormal expression of miRNAs represents a common feature of various cancers, and can be caused by different mechanisms such as amplification/deletion, chromosomal rearrangements, and epigenetic regulation [7]. Depending on the genes targeted, miRNAs can act either as oncogenes or tumor suppressors. Many studies have shown that the aberrant expression of miRNAs, including miR-21, miR-221/222, miR-181s and miR-34s, play an important role in gliomagenesis [8, 9]. MiRNAs show characteristic expression signatures in various cancers and can profoundly affect cancer cell behavior [10]. Importantly, miRNA profiling represents a powerful tool to accurately differentiate cancers from normal tissue and to classify cancer subtypes. However, a new classification system based on miRNA expression has not been reported in glioma.

Herein, we analyzed the miRNA expression profiles from 82 Chinese primary glioblastoma (pGBM) samples, identifying two distinct subclasses of pGBMs: the temozolomide (TMZ) chemoresistant subtype (TCR subtype) and the TMZ chemosensitive subtype (TCS subtype). The TCR subtype has poor prognosis and exhibits increased chemoresistance to TMZ. Furthermore, we propose a classifier consisting of seven miRNAs (miR-1280, miR-1238, miR-938 and miR-423-5p (overexpressed in the TCR subgroup); and let-7i, miR-93 and mi-151-3p (downregulated in the TCR subtype)). The classifier produced 100% prediction accuracy when assigning internal samples to the matching subtypes. The classifier was validated using an independent cohort containing 78 pGBM samples.

## RESULTS

### Molecular subtypes of in pGBM samples

**Figure 1:**
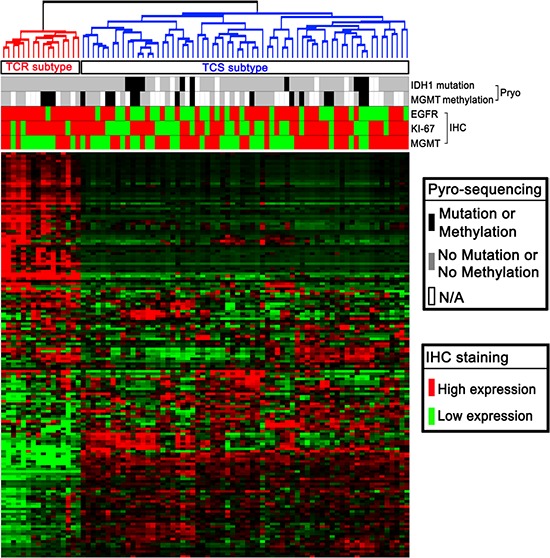
Molecular classification of pGBMs Eight-two Chinese pGBM samples were subjected to whole genome miRNA profiling. Unsupervised clustering using 162 most variable miRNAs identified two main subtypes (TCR and TCS).

In our present study, we obtained miRNA expression profiles for 82 Chinese pGBMs. After data filtering, unsupervised clustering was performed using 162 miRNAs (Supplementary Table 1) that demonstrated highly variable expression across samples (MAD > 0.5) and were associated with survival (Univariate Cox Regression: *p* < 0.1). As shown in Figure 1, unsupervised analysis revealed two distinct subgroups of pGBMs (the TCR and TCS subtypes). Flow chart indicating the carryout of the present study was shown in Supplementary Figure 1.

### Accurate diagnosis of TCR subtypes using expression signatures of seven miRNAs

**Figure 2:**
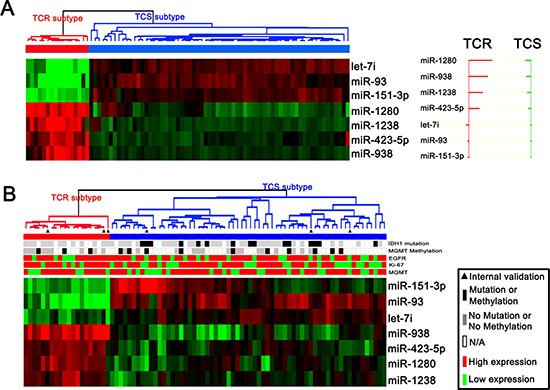
Validation of TCR and TCS subgroups in an independent cohort **(A)** PAM identified a classifier containing seven miRNAs that could clearly differentiate TCR and TCS samples in the 82 samples with miRNA microarrays. **(B)** The classifier could effectively reveal TCR and TCS subtypes in internal and independent validation samples.

An objective classification scheme for clinical applications was then developed by identifying a classifier that can reliably assign an unknown tumor to the defined subtypes. PAM was applied to all the prediction data sets, resulting in a classifier set comprising seven unique miRNAs (miR-1280, miR-1238, miR-938 and miR-423-5p (overexpressed in the TCR subtype), and let-7i, miR-93 and miR-151-3p (downregulated in the TCR subtype). The classifier produced 100% prediction accuracy when assigning samples to the matching subtypes in a 10-fold cross-validation (Figure 2A). We further validated the classifier using 84 pGBM samples. Among the 84 samples, 78 were new independent samples that had not been used for the bead-based expression assay, whereas the remaining six were included in the miRNA profiling assays and were used for internal validation. Expression profiling of the seven miRNAs in the 84 GBM samples was determined by TaqMan quantitative real-time PCR. Data are presented as ΔCt, which refers here to the difference in threshold cycles for an miRNA and U6 RNA. Expression data were mean-centered. Unsupervised average linkage hierarchical clustering was performed. The internal and independent validation samples were clearly assigned into TCR and TCS subgroups (Figure 2B).

### Clinical characteristics of TCR and TCS subtypes

**Figure 3:**
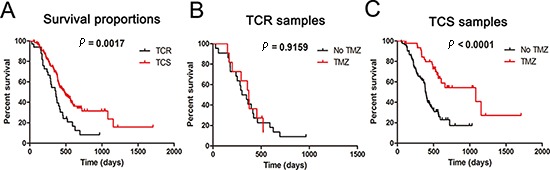
Clinical outcomes of TCR and TCS subgroups **(A)** Kaplan–Meier survival plots for all TCR and TCS samples. **(B)** Kaplan–Meier survival plots for samples treated or not treated with TMZ in TCR samples. **(C)** Kaplan–Meier survival plots for samples treated or not treated with TMZ in TCS samples.

Among the samples used in our study, 33 GBM samples were assigned to the TCR subtype, and 127 cases were assigned to the TCS subtype. In addition, the clinical outcome and genetic alterations of TCR and TCS were analyzed systematically. As shown in Figure 3A, in all samples, TCR was associated with a significantly poorer clinical outcome than TCS samples (*p* = 0.0017). Samples in the TCR group were chemoresistant to TMZ (Figure 3B; *p* = 0.9159). Samples in TCS group were chemosensitive to TMZ (Figure 3C; *p* < 0.0001). As shown in Table 1, samples in the TCR subtype showed no *IDH1* mutation, and showed high expression of Ki-67 and EGFR. TCS samples displayed the opposite results. Age at GBM diagnosis, *MGMT* promoter methylation and protein expression were not different between the TCR and TCS groups.

**Table 1:** Clinical and molecular pathology features of TCR and TCS subtype

	TCR subtype	TCS subtype	*p* value*
**No. of cases**	33 cases (20.6%)	127 cases (79.4%)	
**SEX (Female/Male)**	14/19	47/80	0.688
**Age at GBM diagnosis (year)**	46.9 ± 13.8	45.6 ± 12.5	0.5884^‡^
**IDH1 mutation (Mutated/Unmutated)**	0/26	23/66	0.002
**MGMT promoter methylation (Methylated/Unmethylated)**	6/14	23/39	0.604
**MGMT (Low/High)**	13/20	48/79	1.000
**Ki-67 (Low/High)**	7/26	50/77	0.066
**EGFR (Low/High)**	6/27	46/81	0.060

* Two-sided χ^2^ test.

‡ Student's *t*-test.

## DISCUSSION

The use of gene expression data from patient tumor samples to classify them and to determine better treatment options is becoming increasingly common in clinical practice [13]. The instability of mRNA makes expression profiling a challenging endeavor in routine clinical practice outside of major hospitals or commercial laboratories [14]. The urgent need for an objective, molecular-based classification of GBM is exemplified by the high rate of divergent diagnoses, the inexact prognostic capabilities, and poor therapeutic predictive properties of the current histopathological classification schemes. Here, we profiled miRNA expressions in 82 pGBMs, and unsupervised analysis revealed two main types: the TCR and TCS subtypes. Furthermore, different clinical characteristics of the two subtypes of GBM were defined.

Although the overall prognosis of pGBMs is dismal, the clinical characteristics of pGBMs are very different from each other [15, 16]. Classification based on molecular phenotype may explain the clinical heterogeneity of GBM. Past glioma classification schemes have used mRNA expression data with varying success, and with only moderate concordance between studies. Recently, Kim *et al* reported a developmental taxonomy of GBM defined and maintained by miRNAs [17]. In the present study, two clear subtypes of pGBMs (TCR and TCS) were defined and clinical analysis showed that the TCR subtype has poor prognosis and exhibits increased chemoresistance to TMZ. The TCR subtype is characterized by no *IDH1* mutation, and by EGFR and Ki-67 overexpression. The TCS subtype showed the opposite situation. These results demonstrated new subtypes related to prognosis and chemoresistance, and provide a new direction for future molecular pathological diagnosis and prognosis prediction in GBM.

In the present study, the Human v2.0 miRNA Expression BeadChip was used for whole genome miRNA profiling, in which more than 800 miRNA were detected. Furthermore, a classifier containing seven miRNAs that could clearly differentiate TCR and TCS samples was identified. Then we tried to validate our miRNA classification in The Cancer Genome Atlas (TCGA) samples. However, the Agilent 8 × 15 K Human miRNA microarray used in TCGA only contained 470 miRNAs. Only two of the seven miRNAs in the classifier for diagnosing TCR and TCS subtypes are in the TCGA miRNA dataset. Thus, the TCGA miRNA dataset could not be used as the validation cohort.

TMZ has been approved for the treatment of GBM. Further clinical trials have been performed to assess the activity of TMZ in GBM. *MGMT* promoter hypermethylation is a classical marker for TMZ chemoresistance [18]. Notably, not all patients with GBM having *MGMT* promoter methylation respond to TMZ [19]. In the present study, survival analysis showed that the TCR subtype was TMZ-resistant, while TCS subtype was chemosensitive to TMZ. This pointed out that individualized therapy with TMZ based on above classification should be considered when treating GBM patients. Meanwhile, *MGMT* promoter methylation and protein expression showed no difference between TCR and TCS samples. These results indicated that the chemoresistance of the TCR samples might be caused by a different mechanism associated with aberrant miRNA expression. However, the underlying molecular mechanism remained to be further investigated.

In summary, we identified, for the first time, a novel TMZ-chemoresistant subtype of pGBMs based on miRNA profiles. The new classification system provides a predictor of prognosis and chemosensitivity to TMZ and hints at another mechanism of TMZ resistance. This may help in determining individualized therapy of pGBM patients.

## MATERIALS AND METHODS

### Patients and samples

One hundred and sixty pGBM cases from the Chinese Glioma Genome Atlas (CGGA) were included in this study. All the patients underwent surgical resection between January 2006 and December 2008, and subsequently received radiation therapy and/or alkylating agent-based chemotherapy. Patients were eligible for the study if their diagnosis was established histologically by two neuropathologists, according to the 2007 World Health Organization classification guidelines. Tumor tissue samples were obtained by surgical resection before patients underwent radiation and/or chemotherapy treatment. The institutional review boards of all the hospitals involved in the study gave their approval, and all patients gave written informed consent.

### RNA extraction

All the tissue samples were immediately snap-frozen in liquid nitrogen after surgery. A hematoxylin and eosin-stained frozen section was prepared to assess the percentage of tumor cells before RNA extraction. Only samples with greater than 80% tumor cells were selected. Total RNA from frozen tumor tissues was extracted by using the mirVana miRNA Isolation kit (Ambion, Austin, TX, USA), according to the manufacturer's protocol. RNA concentration and quality were measured using a NanoDrop ND-1000 spectrophotometer (NanoDrop Technologies, Houston, TX, USA).

### MiRNA expression profiling

Eighty-two glioma tissues were randomly chosen for miRNA microarray assays (GSE25632) [11]. Briefly, 200 ng of total RNA was polyadenylated and then converted to cDNA using a biotin-labeled Oligo dT primer with a universal PCR sequence. After cDNA synthesis, miRNAs were individually amplified using specific oligonucleotides. A single miRNA-specific Oligo (MSO) was designed against each mature miRNA sequence, and miRNA-specific primers were extended using DNA polymerase. Universal primers were used to amplify the cDNA templates and the primer complimentary to the array was fluorescently labeled. Finally, the labeled, single-stranded PCR products were hybridized to the Human v2.0 miRNA Expression BeadChip (Illumina Inc., San Diego, CA, USA), which contains 1,146 human miRNAs (97% coverage of the miRBase 12.0 database).

### Quantitative RT-PCR

Real-time RT-PCR was performed using a standard TaqMan PCR kit procedure on a LightCycler 480 real-time PCR system (Roche). All primers and probes for TaqMan microRNA assays were purchased from Applied Biosystems. Real-time RT-PCR was carried out according to the manufacturer's recommendation and the relative expression was calculated using the comparative Ct method.

### Molecular analyses

The *IDH1* mutation and *O6-methylguanine-DNA methyltransferase (MGMT)* promoter methylation were analyzed by pyro-sequencing, as described in our previously report [12].

### Tissue microarray and immunohistochemistry

The tissue microarray was constructed with 160 pGBMs using a manual tissue arrayer (MTA-1; Beecher Instruments, Sun Prairie, WI), and used for immunohistochemical staining of Ki67, epidermal growth factor receptor (EGFR), and O6-methylguanine-DNA methyltransferase (MGMT; Santa Cruz Biotechnology, Santa Cruz, CA), according to the manufacturer's instructions. Appropriate positive and negative controls were run concurrently. Two pathologists, without knowledge of clinical information, jointly scored the staining intensity.

### Subtype identification and prediction

The expression data for the miRNAs were mean centered, and the standard deviation was normalized to 1 per array. We then filtered those with low variability in expression level (median absolute deviation (MAD) < 0.1). We used two additional criteria to further select a group of highly informative miRNAs. These criteria included (i) miRNAs showing highly variable expression (MAD ≥ 0.5; *n* = 66), (ii) patient survival-related miRNAs (0.5 > MAD ≥ 0.1; Univariate Cox model *p* < 0.1; *n* = 96). The resultant 162 miRNAs were used for unsupervised clustering, which was performed and viewed using Cluster 3.0 and Treeview. The diagnostic classifier was identified by prediction analysis of microarrays (PAM).

### Statistical analysis

Kaplan–Meier survival analysis was used to estimate the survival distributions, and the log-rank test was used to assess the statistical significance between stratified survival groups, using GraphPad Prism 5.0. Student's test was used to determine significant differences. Qualitative variables were analyzed using χ^2^ test in SPSS 13.0. All data are presented as the means ± SE. A two-sided P value of < 0.05 was regarded as significant.

## SUPPLEMENTARY FIGURE AND TABLE




