# Supplementary material for: MicroRNA profiling of Chinese primary glioblastoma reveals a temozolomide-chemoresistant subtype
Source: Oncotarget. 2015 Mar 23;6(13):11676–82. doi: 10.18632/oncotarget.3258 (PMC4484485; doi:10.18632/oncotarget.3258)
Supplement: Supplementary file 1 [file oncotarget-06-11676-s001.pdf]

SUPPLEMENTARY FIGURE AND TABLE

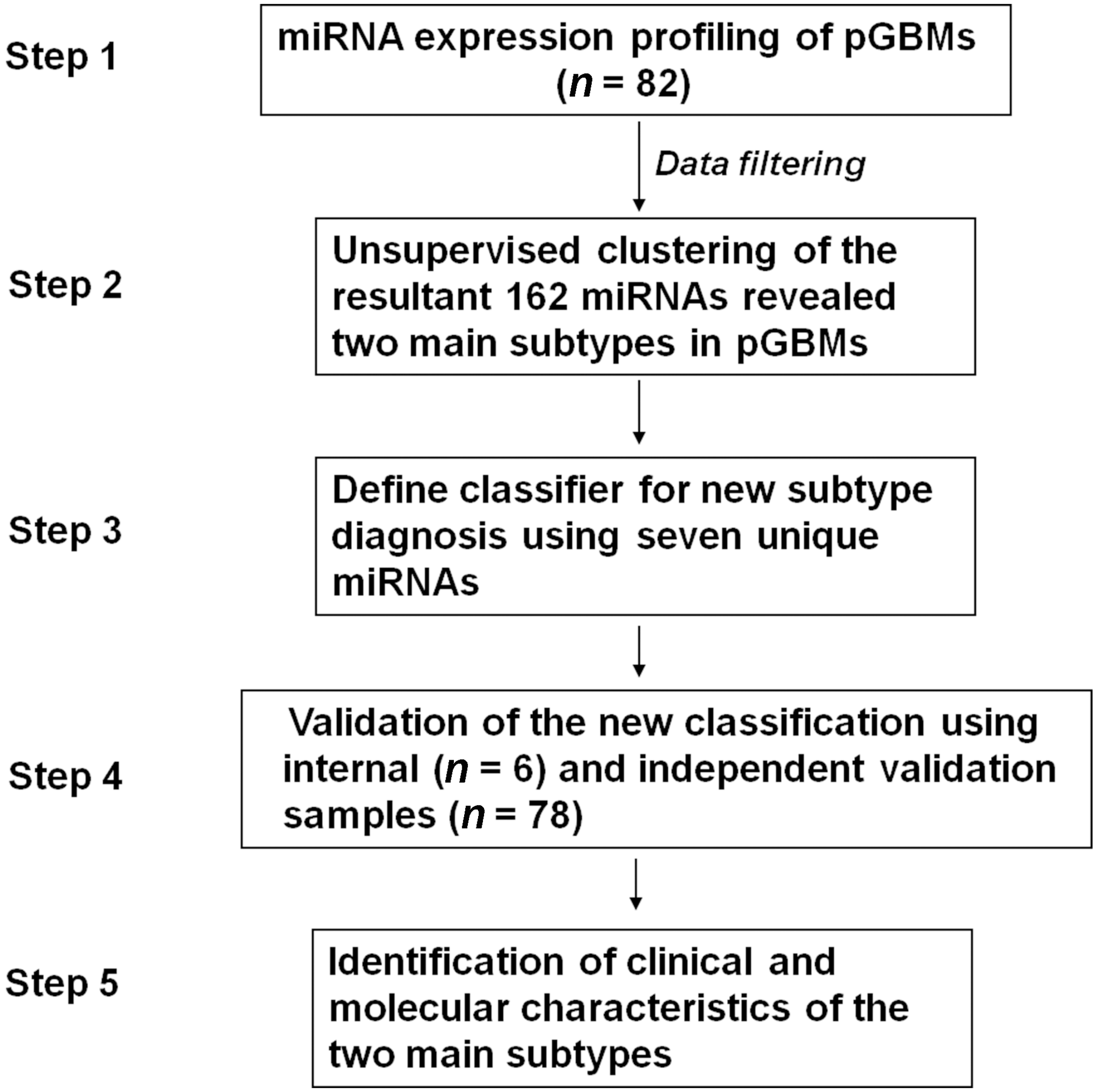

Supplementary Figure 1: Flow chart indicating the carryout of the present study.
